# Childhood Obesity and the Cryptic Language of the Microbiota: Metabolomics’ Upgrading

**DOI:** 10.3390/metabo13030414

**Published:** 2023-03-11

**Authors:** Alice Bosco, Michele Loi, Giulia Pinna, Roberta Pintus, Vassilios Fanos, Angelica Dessì

**Affiliations:** Department of Surgical Sciences, University of Cagliari and Neonatal Intensive Care Unit, AOU Cagliari, 09124 Cagliari, Italy

**Keywords:** metabolomics, obesity, microbiota

## Abstract

The growing obesity epidemic in childhood is increasingly concerning for the related physical and psychological consequences, with a significant impact on health care costs in both the short and the long term. Nonetheless, the scientific community has not yet completely clarified the complex metabolic mechanisms underlying body weight alterations. In only a small percentage of cases, obesity is the result of endocrine, monogenic, or syndromic causes, while in much more cases, lifestyle plays a crucial role in obesity development. In this context, the pediatric age appears to be of considerable importance as prevention strategies together with early intervention can represent important therapeutic tools not only to counteract the comorbidities that increasingly affect children but also to hinder the persistence of obesity in adulthood. Although evidence in the literature supporting the alteration of the microbiota as a critical factor in the etiology of obesity is abundant, it is not yet fully defined and understood. However, increasingly clear evidence is emerging regarding the existence of differentiated metabolic profiles in obese children, with characteristic metabolites. The identification of specific pathology-related biomarkers and the elucidation of the altered metabolic pathways would therefore be desirable in order to clarify aspects that are still poorly understood, such as the consequences of the interaction between the host, the diet, and the microbiota. In fact, metabolomics can characterize the biological behavior of a specific individual in response to external stimuli, offering not only an eventual effective screening and prevention strategy but also the possibility of evaluating adherence and response to dietary intervention.

## 1. Introduction

Globally, childhood obesity represents one of the most important public health problems, and its prevalence, as well as its complications, especially in recent decades, is increasing, particularly in industrialized countries [1,2,3,4,5]. In fact, from 1975 to 2016, the prevalence rates of overweight or obese children and adolescents worldwide increased by more than four times, from 4% to 18%. In 2020, the WHO reported 39 million overweight or obese children under the age of 5 and 340 million between 5 and 19 years [6]. This “pandemic” is strongly associated with important social costs [7], both immediate and in adulthood, since obese children will tend to become obese adults [8,9,10]. The former is due to the significant indirect costs caused by the increased risk of psychosocial problems in children [11], bullying, and absences from school, with consequent poor school results [7]. Furthermore, childhood obesity increases the risk of parental absence from work due to related complications [12], resulting in significant productivity losses [13]. In the United States alone, the incidence in preschool children is 12.3%, 11.8%, and 2% for overweight, obesity, and severe obesity, respectively, rising to 19.4%, 8.9%, and 10.1%, respectively, in adolescents [14]. In addition to the economic and psychosocial problems of stigmatization and discrimination [15] deriving from weight gain, childhood obesity can increase the risk of the onset of a series of morbid conditions. The most common [16] metabolic disorder and the first to be diagnosed in obese children is insulin resistance (IR) [17], representing an important link with type 2 diabetes mellitus (T2DM) [18] and cardiovascular disease [4]. The evaluation of IR is, still today, being discussed in the pediatric field, since most of the proposed indices and diagnostic criteria are based on those of adults, and their application in children is not unanimously accepted [17]. Its identification could therefore be an effective strategy for the prevention and treatment of obesity-related complications [4]. Obesity can also favor the onset of hypertension, dyslipidemia, chronic inflammation, hyperuricemia, nonalcoholic fatty liver disease [19,20], some types of cancer [21,22] and obstructive sleep apnea [23].

However, despite extensive research, the complex molecular basis of body-weight-related metabolic perturbations is not yet fully understood [24,25,26]. In fact, only in a small percentage of cases is obesity the result of endocrine, monogenic, or syndromic causes, while a sedentary lifestyle, urbanization, and changes in eating habits appear to be the main causes [27].

There are now several authors who suggest the need to distinguish between metabolically “healthy” (metabolically healthy obese, MHO) and “unhealthy” (metabolically unhealthy obese, MUO) subjects, even at an early age [28]. Indeed, numerous comorbidities, typical of adults, are also present in children [29], but not all individuals affected by obesity present a similar degree of obesity-related complications [30]. MHO children are obese, but do not show any metabolic complications [31,32], unlike MUOs, and they do not necessarily have lower lifetime morbidity and mortality and may transition to the MUO phenotype during puberty [30]. To date, given the scarcity of data on the subject, universal diagnostic criteria have not yet been defined, and early screening for MHO and MUO could help review current prevention and treatment strategies with a consequent reduction in health care costs [31]. Therefore, a good understanding of the metabolic mechanisms underlying obesity and its complications is inevitable for the development of appropriate screening strategies [33]. In this regard, metabolomics could represent a useful tool to identify some eventual biomarkers, considering the numerous clinical applications of this technique [34,35,36], both from a preventive perspective and to optimize therapeutic strategies. In this context, the correlation with the intestinal microbiota appears essential, the alterations of which, strongly linked to the metabolic profile, have been studied both as a cause and as a consequence of obesity and its complications. Currently, the first steps are being taken towards a universal definition of MHO in children, as the scientific community would seem to agree in defining children with obesity according to body mass index (BMI) by age and gender (BMI z-score) according to the WHO growth chart by classifying those meeting all cardiometabolic criteria as MHO. This certainly represents a first step to limit the variability of definitions and facilitate comparison between studies [30].

## 2. Childhood Obesity

Obesity is a disorder characterized by a disproportionate increase in body weight with respect to height, mainly due to the accumulation of adipose tissue [24], which can lead to impairment of the patient’s physical and/or psychological function [37]. Given the complexity in directly measuring nutritional status, to easily define overweight or obesity, the use of body mass index (BMI) is well established. In fact, this value represents an indirect estimate of body fat, calculated through the ratio between the weight expressed in kilograms and the square of the height in meters. However, in children, absolute BMI is not used as a risk marker because its constituent measures vary as a function of normal growth and maturation, necessitating standardization by age and gender [37]. Therefore, in order to identify the health risks related to excess weight in children and adolescents, age- and gender-specific BMI percentiles, or z-scores, are used, based on reference growth curves, created on the basis of longitudinal and/or cross-sectional studies with samples of children and adolescents considered as standard [38]. The most used are the Centers for Disease Control and Prevention (CDC) (2000) but above all those of the World Health Organization (WHO) (2006/2007) and of the International Obesity Task Force (IOTF) (2012) growth charts. The two main anthropometric indicators used are length/height by age and BMI by age, as they provide two fundamental parameters, such as the growth trajectory and the identification of underweight or overweight conditions [38]. CDC growth charts, based on studies conducted in the United States, are expressed in sex- and age-specific percentiles for children and adolescents aged 2 to 19 years, which delineate overweight as a BMI between the 85th and 94th percentiles (included), while obesity is defined by a value ≥ the 95th percentile [38,39].

The WHO growth diagrams are expressed in percentiles or z-scores and are specific by gender and age group [38]. Although the WHO recommends the use of its growth tables (2006/2007) for international use, some researchers have highlighted some concerns regarding the choice of BMI percentile cut-offs for these curves [29]. Indeed, the most recent curves proposed by the IOFT use a different system, with specific BMI curves, constructed so as to correspond to the values of 25 kg/m^2^ (overweight) and 30 kg/m^2^ (obesity) at 18 years, thus providing BMI cut-offs by age and gender for overweight and obesity, based on large datasets from six countries or regions covering different races/ethnicities. This is possible because the definitions of obesity and overweight at age 18, associated with health outcomes in adults, are considered to be extendable to younger ages.

However, despite the widespread use of percentiles in clinical practice for several years now, the evidence regarding their usefulness is not yet solid, given the need to analyze different pediatric samples. In this regard, some studies show encouraging data, such as those emerging from the analysis of biracial samples of children and adolescents, in supporting the usefulness of the 95th percentile as a useful threshold for predicting high values of visceral abdominal fat and cardiometabolic risk [39]. Conversely, other studies seem to support that the use of BMI does not allow insight into the biology of obesity and its comorbidities.

Nonetheless, the prediction of disease risk, fat mass, and fat-free mass assessed with validated techniques for weight status assessment including detailed phenotyping by body composition analysis (BCA), such as densitometry, dual-energy X-ray absorptiometry, and bioelectrical impedance analysis, does not appear to exceed the BMI value [40].

For these reasons, the study of the concept of functional body composition (FBC) began, which refers to the masses of body components, organs, and tissues, as well as their interrelationships in the context of endocrine, metabolic, and immune functions in order to define specific phenotypes of obesity, such as the sarcopenic-obese patient [40]. The most recent data in this regard, concerning the pediatric population, come from a cross-sectional study [41] on 15,392 children and adolescents aged between 5 and 17 years, in which body composition was assessed by means of bioelectrical impedance using a population-specific algorithm. This was achieved through the use of age- and gender-specific percentiles of BMI, fat mass index (FMI), fat-free mass index (FFMI), and a “weight-bearing model” (characterized by ratios of fat, FM, fat-free mass, FFM, and FM/FFM ratio2) modeled using the LMS method. This method, unlike the percentile curves, which show the distribution of a measure as a covariant variation, frequently age, summarizes the changing distribution through the use of three curves, median, coefficient of variation, and asymmetry [42]. This analysis showed that the prevalence of low FFM relative to FM affected more than 60% of overweight children and adolescents, highlighting an early risk of sarcopenia in these subjects. The authors, therefore, concluded that these pediatric body composition data could represent a relevant reference that may allow clinicians and scientists to more closely monitor body composition during growth and development and to tailor the interpretation of the data [41].

## 3. Obesity and Microbiota in Children

The human intestine is colonized by a large multitude of different microorganisms (intestinal microbiota), mostly bacteria [43], but also by viruses, archaea, and protozoa [8,44]. Although microbial DNA has been identified in samples of placenta, amniotic fluid, and meconium, there is still no unanimous agreement in the scientific community regarding in utero colonization of the fetus [45,46]. In fact, the current evidence-based consensus maintains that healthy newborns are definitely colonized by microbes during and after birth [45,46,47,48] and that this colonization continues during the first years of infancy, reaching, between 3 and 5 years, a stable microbiota similar to that of adults [10]. The development of the microbiota is influenced both by numerous external factors, such as the type of feeding and delivery and the early use of antibiotics [48,49,50], and by characteristic factors of the host [50,51,52]. Therefore, what happens in the first years of life appears fundamental to define its composition.

Generally, a term infant from vaginal delivery is characterized above all by facultative anaerobic bacteria, such as *Escherichia* spp. and other members of the *Enterobacteriaceae* family [53] together with an enrichment of *Lactobacillus*, which is the nucleus of the maternal vaginal microbiota [48]. Then, in the first days of life, colonization by *Bifidobacterium* and *Clostridium* [53] begins. In fact, with the beginning of breastfeeding, the microbiota is enriched and differentiated: higher levels of *bifidobacteria* are observed in breastfed infants, while infants fed with formula milk are characterized by a more diversified intestinal microbiota, dominated by *Staphylococci*, *Bacteroides*, *Clostridia*, *Enterococci, Enterobacteria*, and the genus *Atopobium.* An important maturation occurs following the introduction of complementary feeding with a significant increase in alpha diversity determined by the replacement of *Proteobacteria* and *Actinobacteria* with the phyla *Firmicutes* and *Bacteroidetes* [54] and a high increase in the production of short-chain fatty acids (SCFAs) [54].

However, although the introduction of solid feeding results in a substantial change in the relative abundances of the infant’s gut microbiota and also the related microbial transcriptome, it has been shown that the fecal microbiota profile assessed at 3 months of age (mainly composed of *Bacteroidaceae, Bifidobacteriaceae, Enterobacteriaceae, Lachnospiraceae, Ruminococcaceae*, and *Veillonellaceae*) would be a more reliable predictor of future risk of overweight than the analysis of the 12-month microbiota profile [54]. In support of this, there is a meta-analysis conducted on over 200,000 participants, which showed a statistically significant reduction in the risk of obesity (pooled adjusted OR: 0.78; 95% CI: 0.74–0.81) in breastfed children, from which evidence also emerges to support a dose–response relationship between the duration of breastfeeding and a reduction in the risk of obesity [55].

To date, there is now numerous evidence regarding the strong influence of the microbiota on numerous physiological processes and on host behavior [56,57] as well as its correlation with various pathologies also inherent in the pediatric population, such as nosocomial infections, inflammatory bowel disease (IBD) [58,59], obesity [60,61], insulin resistance, and allergies [55,62,63].

The first data on the role of the microbiota in obesity derive from studies conducted on animal models where fecal transplantation showed that the microbiota from an obese donor promotes weight gain in germ-free recipients with the same diet, proving that the microbiota itself may be obesogenic [64]. Indeed, it would seem that microbiota actively participate in the development and maintenance of weight gain by modifying eating behavior, calories absorbed from food, energy metabolism, and fat deposition. Various mechanisms have been proposed in the literature to explain the relationship between the composition of the intestinal microbiota and the development of obesity; however, the evidence in this regard is still contradictory and sometimes inconclusive also due to methodological shortcomings of the studies, both on humans and in the animal model.

The main alterations observed in the studies conducted so far are multiple and affect the metabolic and inflammatory state together with the hormonal profile, even if the presence of any causal relationship has not yet been ascertained [65,66].

The main involved metabolic mechanisms concern a greater ability to extract energy from nondigestible polysaccharides by intestinal microbes [64], with the production of SCFAs and a consequent modification of food absorption. At the metabolic level, a decrease in the ability to oxidize fatty acids in the muscle mediated by the decrease in AMP kinase, a sensor of the cellular energy state; an increased hepatic lipogenesis via ChREBP/SREBP-1; and an alteration of the metabolism of bile acids, which affects the correct transport of cholesterol, were also observed [64].

As far as the hormonal structure is concerned, there are several mechanisms through which the intestinal microbiota with the production of SCFAs can influence not only the energy metabolism of the host but also its eating behavior with important repercussions on the development of obesity [65]. For instance, there is glucagon-like peptide-1 (GLP-1), responsible for the delicate regulation of communication between the nutritional load in the intestinal lumen and peripheral organs, such as the brain, liver, muscle, and adipose tissue. Its regulatory activity is carried out through the postprandial increase in satiety and intestinal transit time together with the incretin effect, which determines a greater secretion of insulin. The production of GLP-1 is influenced by the intestinal microbiota through multiple mechanisms: an action on the regulation of the expression of its precursor, proglucagon; an increase in GLP-1-positive enteroendocrine L cells in the intestine; or the activation of GLP-1 receptors G protein-coupled 43 and 41 (GPR41 and GPR43), expressed by intestinal epithelial cells, endocrine cells, and adipocytes. Furthermore, mechanisms involving the stimulation of GPR41-coupled receptors contribute to the production of the intestinal anorexigenic hormone, peptide YY (PYY), with an important contribution in the regulation of satiety, a decrease in gastric emptying and intestinal transit time, and increased energy harvest and hepatic lipogenesis. In addition, GPR43s in white adipose tissue act as sensors of postprandial energy excess, regulating energy expenditure [65,66].

A further correlation between the microbiota and host energy metabolism is mediated by fasting-induced adipocyte factor (FIAF). In fact, the inhibition of FIAF by the intestinal microbiota determines the lack of inhibitory action on lipoprotein lipase (LPL), responsible for the accumulation of fat in peripheral tissues [65].

Regarding the microbial component in the pathophysiology of obesity, there is still no solid evidence regarding the extent of the variation in the relative abundance of the intestinal microbiota in children. Indeed, as shown in the 2018 systematic review by Indiani et al. [67], although changes in the levels of the phyla Firmicutes and Bacteroidetes could be a significant factor in childhood obesity, the limited number of papers evaluating these phyla entirely and the heterogeneity among the species evaluated do not allow us to draw certain conclusions.

As regards the phylum *Firmicutes*, Gram-positive microorganisms, their correlation with obesity emerges despite the presence of some contradictory results, probably determined by the different species involved in each investigation [67]. From the literature, it emerges that the increase in the species *C. leptum* and *E. hallii*, together with the decrease in *Faecalibacterium prausnitzii* and *C. difficile*, has been associated with obesity/overweight in infants and children in preschool/school age [67]. Among the possible mechanisms involved, there is a lack of anti-inflammatory effect associated with the lack of *F. prausnitzii* and the high fermentative capacity of sugars and nonabsorbable fibers by *C. leptum*, with consequent production of high quantities of SCFAs [67]. Although with a lesser degree of evidence, an association (species dependent) emerges between Lactobacillus spp. (LB) and a higher BMI, while the Staphylococcus genus was correlated with lower BMI [67].

With regard to the Bacteroidetes phylum, predominantly Gram-negative microorganisms, the systematic review of the literature [67] shows a positive association between *B. fragilis* and obesity in children with a high/moderate degree of evidence. However, the causal mechanism related to the inflammatory stimulus due to the presence of LPS in the outer membrane of Gram-negatives, such as *B. fragilis*, needs further investigation. Other Gram-negative bacteria belonging to the phylum Bacteroidetes, such as *Bacteroides* and *Prevotella*, were found to be less abundant in obese subjects, although they have been associated with intestinal inflammation, highlighting the need for specific species investigations [67].

Nonetheless, the overall analysis of the entire Bacteroidetes phylum shows a significant reduction in their levels and, consequently, in the *Bacteroidetes/Firmicutes* ratio when obese children were compared with control children [67].

In recent years, further studies have been performed. López-Contreras et al. [68] did not show significant differences in the abundances of the phyla or in the *Firmicutes/Bacteroidetes* ratios between normal weight and obese children. However, the analysis of single species has highlighted a different trend in obese children. In fact, the presence of higher concentrations of *Bacteroides eggerthii* in this population and a positive correlation with the percentage of body fat, which became negative with fiber intake, emerged. On the contrary, the 16S profile by Gallardo-Becerra et al. [69] confirmed a higher abundance of *Firmicutes* and a decrease in *Bacteroidetes* in the obesity groups, as well as a significantly higher richness and diversity than in the normal weight group. Further confirmation of the importance of the microbiota on the development of obesity comes from the evidence that the use of antibiotics in childhood leads to an increased risk of developing this pathology [70,71], in particular if exposure takes place in the first years of life [72]. In fact, microbiota studies have shown that after repeated exposure to antibiotics, newborns show a reduction of potentially antiobesogenic bacteria (*Bifidobacteria* and *Bacteroides*) [73].

In 2022, Houtman et al. [74] investigated whether in healthy children differences in the *Firmicutes /Bacteroides* ratio can be detected in childhood (during the first 12 years of life) in relation to the BMI z-score and whether there is a relationship between SCFA-producing bacteria and obesity. However, exploratory analyses conducted with multilevel modeling and a random forest algorithm suggest that the relative abundances of these two phyla were independently negatively associated with BMI z-score from infancy through childhood, and that the SCFA-producing genera *Subdoligranulum* and *Alistipes* are states negatively correlated with future BMI in childhood.

## 4. Microbiota–Gut–Brain Axis in Children

Evidence regarding the important contribution of the human gut microbial population in regulating behavior and brain function is increasingly compelling. In fact, there is an important cross-talk between the microbiota and the brain, the so-called microbiota–gut–brain axis. The mechanisms underlying this delicate interaction mainly involve the vagus nerve, the immune system, the neuroendocrine pathways, and the metabolites of bacterial origin [75,76,77]. The vagus nerve, in its afferent branch, is the main neural duct, which, in mammals, connects the gastrointestinal tract to the nucleus of the solitary tract and to the complex emotion regulation network, which, although in the absence of direct interaction with the intestinal microbiota, is influenced by it. Indeed, vagal afferences perceive microbial signals, both in the form of bacterial metabolites and through the microbial modulation of the activity of enteroendocrine intestinal cells (EECs), including enterochromaffins (ECCs). This activity is perceived directly through the action of SCFAs, via the free fatty acid receptors (FFARs), but also through the mediation of enteroendocrine cells, via the release of serotonin, which activates the 5-hydroxytryptamine-3 receptors in the afferents’ vagal fibers or by other intestinal hormones [76,77].

The immune system contributes to completing this cross-talk complex, which is fundamental not only in maintaining the balance between the homeostatic tolerance of commensal organisms and the contemporary protection of the organism from the invasion of pathogenic microbes, but also in the mediation between the microbiota itself and the central nervous system (CNS). Indeed, in case of imbalance, a simple local immune activation can affect the proper permeability of epithelial tight junctions, resulting in the release of mediators into the systemic circulation with the subsequent eventual metabolic endotoxemia, which can cause immune activation in different organs, including the brain. Furthermore, the toll-like receptor (TLR), together with other components of the innate immune system, acting as sensors of the intestinal microbial presence, can communicate with the intestinal nervous system (ENS), determining changes in its development and function, although the way in which microbe-TLR communication influences the structure and function of the ENS has not been determined yet [76,77].

As far as microbial metabolites are concerned, they appear directly involved in the modulation of the CNS and ENS. In fact, through SCFAs, a regulation of intestinal and cerebral motility, secretion, and signaling is achieved by FFARs on epithelial cells, EECs, ECCs, immune cells, and intrinsic and extrinsic neurons together with a regulation of the expression of enzymes involved in the biosynthesis of neurotransmitters in the brain [76,77]. The evidence regarding the key contribution of serotonin in the signaling of the microbiota–intestine–brain axis, which in fact acts as a neurotransmitter both in the CNS and in the ENS, is now solid. In fact, most of the production of serotonin occurs by the enterochromaffin cells of the gastrointestinal epithelium, where it acts as a paracrine hormone. To this is added an endocrine action, thanks to the transport in the blood circulation mediated by platelets, which determines its systemic effects [78]. Recent studies on animal models support bidirectional effects of this neurotransmitter in the microbiota–gut–brain axis. In fact, it would not be only the production of serotonin that exerts central effects through the vagal afferences, but a local action would also seem to occur, through which the concentrations of this neurotransmitter would favor the colonization of specific species [78].

In addition to this, the contribution of the microbiota is also achieved through the regulation of the availability of neurotransmitter precursors, which therefore also affects the availability of tryptophan, the only precursor for the biosynthesis of serotonin. In the host, the kynurenine pathway represents the major metabolic pathway responsible for regulating tryptophan availability. This metabolic pathway is mainly initiated by the induction of the enzyme indoleamine-2,3-dioxygenase (IDO), localized in the brain, gastrointestinal tract, and liver, or by tryptophan-2,3-dioxygenase (TDO), almost exclusively expressed in the liver. The activation of this metabolic pathway, especially regarding the action of IDO, can occur in response to immune stimuli, such as interferon-gamma. Kynurenine is then obtained from tryptophan, which then undergoes further metabolization, through two possible routes, that of kynurenic acid (KYNA) and that of quinolinic acid (QUIN). KYNA and QUIN are globally referred to as “kynurenines” and are able not only to mediate inflammatory stimuli but also to cross the blood–brain barrier (BBB) to reach the CNS. However, their metabolic role is opposite, while KYNA is a neuroprotective N-methyl-D-aspartate (NMDA) receptor antagonist, QUIN represents a neurotoxic NMDA receptor agonist. Therefore, the imbalance between the neurotoxic and neuroprotective properties of kynurenines may play a delicate role in functional brain disorders [79]. In this context, studies on the animal model have shown how the stimulation and inhibition of the kynurenine pathway is related to the microbiota itself and to the production of particular metabolites, such as hydrogen peroxide, by the lactobacilli, which acts as an inhibitor of IDO, with behavioral implications [80].

Other metabolic pathways of tryptophan were found to be relevant for the microbiota–gut–brain axis. Among these, important evidence has emerged regarding the microbial processing of tryptophan into indole, thanks to the ability of these metabolites to act as ligands for arylhydrocarbon receptor activation (AhR). Indeed, they cross the BBB to activate AhR in astrocytes and microglial cells, resulting in the suppression of proinflammatory NF-kB signaling [77].

Tryptamine, a further bacterial metabolite of tryptophan, has attracted interest due to its ability to act through G protein-coupled receptors, such as 5-HT4R, to influence host physiology. However, to date, it is still not clear whether tryptamine of microbial origin can reach the central nervous system [76].

Moreover, as regards the bacterial production of specific neurotransmitters, such as norepinephrine, dopamine, and GABA, given their short half-life and reduced ability to cross the blood–brain barrier, there is still no evidence regarding the possibility of reaching, in relevant concentrations, specific sites inside the CNS [76].

Thus, it is inferred that the alteration of the gut microbiota may lead to obesity precisely through modulation of the gut–brain axis, influencing physiological function and behavior.

## 5. Metabolomics in Childhood Obesity

**Figure 1:**
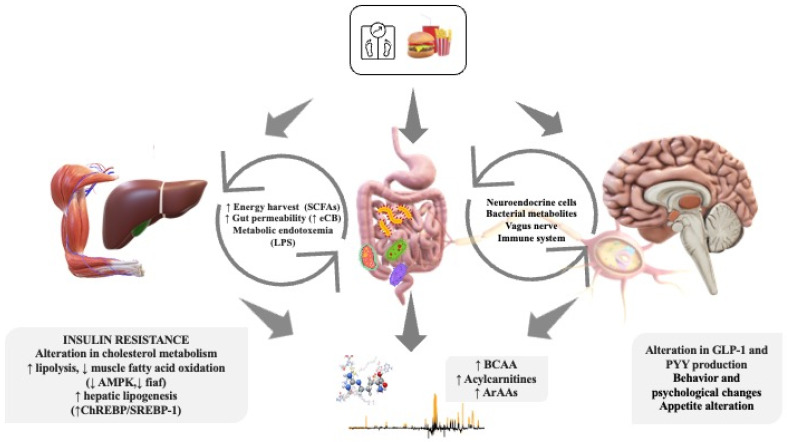
Connection between diet, gut microbiota, and metabolomics in childhood obesity.

To better characterize the “specific phenotype” of obese children, together with data on the microbiome, one can try to identify the “metabolomic fingerprint”. Metabolomics is a science that studies small molecules (<1500 kDa), such as nutritional intermediates, hormones, and other signal molecules, in tissues and biological fluids [19]. It provides an immediate snapshot of the entire physiology of an organism and allows for the identification of metabolites potentially involved in disease mechanisms by detecting changes in the level of numerous analytes in individuals with disease compared with healthy ones [81]. It may therefore help define the characteristic molecular phenotypes and better elucidate the complex pathological mechanisms of obesity [82] and the correlation between obesity and metabolic diseases [83]. It is a science whose techniques and applications are growing exponentially [84]. Indeed, it has the advantage of being very rapid (it detects variations that occur in seconds instead of minutes or hours), simple, and with a wide diagnostic potential (detects changes in the concentration of numerous metabolites dynamically over time) [85]. The timeliness and optimization of the diagnosis are among the most important objectives of this technology [84]. In fact, the literature shows how metabolomics represents a very promising tool in the early diagnosis of various fetal, perinatal, pediatric, and adult conditions, thanks to the possible identification of specific and sensitive biomarkers [85], allowing risk stratification and close disease monitoring [86,87]. Such science, therefore, has the potential to aid decision making in the field of personalized medicine [88,89,90]. Therefore, the identification of possible biomarkers in pediatric populations via metabolomics could provide an opportunity not only to better characterize this condition, but also to find new and more effective prevention and treatment approaches [91], allowing for tailor-made management [85,92]. The close correlation between microbiota and metabolomic analysis is schematized in Figure 1.

In this context, the studies present in the literature are increasing: in fact, two systematic reviews have recently been published [26,86].

It is possible to categorize the studies conducted up to now according to the anomalies found, through a pathway analysis, where the recurring metabolites are classified into broader classes, such as amino acids, lipids, and carbohydrates, within which it is possible to place the metabolic pathway presumably altered, then trying to correlate the different alterations observed with each other [26].

Another possible classification concerns the aims of the research. In fact, through transversal studies, the most represented in this area, it is possible to describe the metabolomic signature of obesity as the metabolic profile of the subject in the presence of the disease is outlined. Conversely, longitudinal studies elucidate the metabolomic profile before the onset of excess fat (children who later developed obesity vs. children who remained normal weighted) in an attempt to identify specific metabolites for risk prediction, while intervention studies evaluate the metabolome in relation to a lifestyle intervention (reduction of BMI), thus highlighting the possible reversibility of the pathology with a view to optimizing therapeutic pathways [86].

From the pathways analysis conducted in the systematic review by De Spiegeleer et al. [26], with a reproducibility considered acceptable by the authors, the centrality of insulin resistance (IR) also emerges in obesity in childhood, despite the absence of measures of internationally agreed reference in children. In fact, a marked increase, albeit inefficient, in the use of carbohydrates and fatty acids was found, attributable to the cell membrane hypothesis of IR and to mitochondrial toxicity. This is due to the fact that the metabolism of glucose and lipids is closely related to mitochondrial function, and therefore, when the oxidation of nutrients is inefficient, and the ratio between ATP production and oxygen consumption is low, there is an increase in the production of superoxide anions with the stimulation of proinflammatory processes [93]. Thus, there is evidence of initial impairment of tricarboxylic acid (TCA) cycle flux and ß-oxidation, resulting in a shift of carbon metabolism towards hypoxic conditions. The elevated values of branched amino acids (BCAA) observed were also involved in the alterations of ß-oxidation, in the stimulation of gluconeogenesis, in the reduced ketogenesis, and in the alteration of the permeability of the gastrointestinal membrane. According to the authors, these metabolic characteristics could allow an initial differentiation of obese children with IR from those without IR. In fact, Mastrangelo et al. [17] and Martos-Moreno et al. [94] deduced that prepubertal children with obesity and insulin resistance have an increase in BCAAs compared with obese prepubertal children without insulin resistance. The predictive value of BCAAs for insulin resistance was already highlighted in nondiabetic adolescents by Tricò et al. [95].

The etiology of such obesity-related increases remains incompletely understood. One possibility is that obese individuals take higher amounts of BCAAs, and in the particular context of a high-fat diet, typical of diet-induced obesity, it could be a further contributor to insulin resistance [87,96]. Specifically, leucine and isoleucine, together with glutamate, are insulin secretagogues, and therefore, their chronic increase, enhancing insulin secretion, could contribute both to hyperinsulinism and, consequently, to pancreatic beta cell insufficiency [96]. In addition, glutamate can cause direct toxicity to pancreatic beta cells [97]. Finally, insulin resistance, in turn, may determine the failure of the physiological ability of insulin itself to suppress BCAA levels [98]. According to some authors, the activation of this pathway alone may not be sufficient to cause IR and the consequent complications, but it could still represent an important factor contributing to both inflammation and hyperinsulinemia [4]. In fact, the possibility is outlined that the toxic metabolites deriving from an altered metabolism of the BCAAs are the main ones responsible for the IR. According to this model, the increase in BCAAs resulting from diet, low rate of catabolism in adipose tissue, or insulin-induced proteolysis favors their catabolism in the liver and skeletal muscle. This leads to the accumulation of some by-products, such as ketoisocaproic acid, short-chain acylcarnitines, and their intermediates, which impair the oxidation of glucose and fatty acids, producing substrates that are not fully oxidized, involved in mitochondrial stress and impaired insulin signaling [4].

Other authors point to the still poor awareness of the mechanisms linking IR and BCAAs, hypothesizing that BCAAs may also cause IR through the action of the mammalian target of rapamycin complex 1, mTORC1, or simply reflect metabolic changes related to IR [86].

Furthermore, from the systematic review of the literature by Handakas et al. [86], it emerges that the most widespread and consistent associations between obesity and metabolomic alterations concern BCAAs, together with some aromatic amino acids (ArAAs), such as tyrosine and phenylalanine. Even in this case, a strong association between these alterations and insulin resistance was highlighted; however, according to the authors, it would be useful to analyze a correlation between these alterations and the concomitant variations of other metabolites.

According to some authors, together with BCAAs, an alteration of acylcarnitine levels could be used to discriminate between children with or without insulin resistance [38]. Acylcarnitines, crucial in the transport of fatty acids to the mitochondria for ß-oxidation [86], may result from incomplete oxidation of fatty acids, and the accumulation of these metabolites, known as lipotoxicity, has been implicated in the development of IR and type 2 diabetes [4]. Newbern et al. [99] found how HOMA-IR in males correlates positively with BMI z-score and with a metabolic signature containing BCAAs, uric acid, and long-chain acylcarnitines. Mastrangelo et al. [18] highlighted that central carbon metabolism (CCM), which includes glycolysis, tricarboxylic acid, and acylcarnitine metabolism, was the most impaired process in obese children with IR, albeit with a small percentage change in the two groups. In fact, most of the statistically significant metabolites that allowed a discrimination between the groups belong to one or more pathways involved in the CCM. Specifically, in addition to the already-discussed increase in BCAAs, obese children with IR showed an overall increase in amino acids, such as alanine, proline, and ArAAs (phenylalanine, tyrosine, and tryptophan); an increase in the levels of ketoisocaproic acid, C3, and C4 acylcarnitines; and a decrease in free carnitine. Handakas et al. [86] associated 17 different acylcarnitines with BMI, reporting an increase in short-chain acylcarnitines, including free carnitine, acetylcarnitine (C2), propionylcarnitine (C3), butyrylcarnitine (C4), valerylcarnitine (C5) and 2-methylbutyrylcarnitine (C5). They highlighted that most of the analyzed studies reported increases in acyl carnitines in addition to BCAAs and that these increases may represent increased availability of acyl-CoA from BCAA catabolism. As regards the longer-chain acylcarnitines, they point out the decrease in oleoylcarnitine (C18:1) in several studies as an index of a reduction in the catabolism of fatty acids. Overall, among the alterations of the lipid metabolic pathway, C3 and C5 acylcarnitines appear to have been detected with the greatest frequency. Since C3 and C5 acylcarnitines are by-products of BCAAs, the reduced complete oxidation of fatty acids appears to be directly affected by BCAA metabolism, supporting an important interaction between amino acid and lipid metabolism [4].

All this could explain the correlation between incomplete fatty acid oxidation and a higher IR score [4], as an incomplete reduction of fatty acid oxidation could lead to a stimulation of the proinflammatory pathways, an ineffective action of the insulin in skeletal muscle, an increase in mitochondrial stress, and consequently impaired blood glucose [4,100].

Furthermore, for some authors, it would be important to differentiate the metabolomic profiles between boys and girls during childhood [37], also on the basis of pubertal age [17]. In fact, some studies show evidence supporting a sex-specific metabolic susceptibility [17,100].

There are also several potential mechanisms that correlate IR with the increase in ArAAs levels, such as the increase in protein degradation, the impairment of an efficient oxidative metabolism in some tissues, or even a reduced de novo synthesis by the intestinal microbiome [86]. In this regard, tyrosine is often included in the “BCAA metabolic signature” associated with obesity and IR even in studies on children [4,26,86]. The most frequent association is represented by an increase in BMI together with an increase in tyrosine [86]. Tyrosine, a neutral aromatic amino acid that results from the hydroxylation of phenylalanine by phenylalanine hydroxylase, is identified in some studies as a potential early marker for the onset of IR [100]. Indeed, the cross-sectional and longitudinal analyses conducted by Hellmutt et al. [101] confirm a relationship between tyrosine and the HOMA index in obese children, whereas BCAA levels were negatively correlated with the IR in the cross-sectional analyses, but in the longitudinal analysis, no significant association was highlighted, not supporting the causal role of BCAAs in inducing IR. In addition, different associations emerged between HOMA and AA in responders to the 1-year lifestyle intervention versus nonresponders, supporting possible different mechanisms for the development of obesity-induced IR.

Overall, from the systematic literature review by De Spiegeller et al. [26], it emerges that in most of the studies, BCAAs and ArAAs, especially tyrosine, were analyzed in combination, showing a similar change trend in obese children, probably due to a competition for the same neutral amino acid transporters in the tissues [4].

Regarding the close correlation with the intestinal microbiota, including the increase in tryptophan and related polyamines, kynurenate and kynurenine, a possible index of immune activation or low-grade systemic inflammation, the increase of which may derive from an upregulation of the IDO activity, was also identified [86].

Another consistent association is represented by the increase in steroid hormones, in particular androgens. In this regard, the condition of obesity has been associated with the timing of puberty onset, particularly for girls. In fact, Perng et al. [102] showed an association between the androgen hormonal pattern and the pubertal characteristics reported by the parents. Given the relationship between the pubertal period and the increased risk of cardiometabolic disorders in adult life, childhood obesity could be linked to a later onset of CVD, thanks to alterations in steroid hormones [26].

Correlations have also emerged with other classes of lipids, given the functional heterogeneity of this class, which extends from the metabolic substrate to the function of cellular signalers, in relation to the length and degree of unsaturation of the chain. Specifically, metabolomics studies have highlighted the presence of multiple associations for fatty acids, long-chain fatty acids, lysolipids, lysophosphatidylcholines (LPC), phosphatidylcholines (PC), and sphingomyelins (SM), confirmed by specific lipidomics studies [86]. The most frequent findings showed a decrease in acyl-alky-PCs, medium-chain-length LPCs, and MS C16:0 [86].

Therefore, there is an altered metabolic profile in obese children who are characterized by a specific metabolomic imprint; however, longitudinal studies, albeit still small, could represent the new frontier in order to reduce the onset of this pathology. A review by Handakas et al. [86] in fact highlighted the main characteristics of this class of studies. They found five studies in the literature, three conducted on cord blood and two on plasma during early childhood. A study by Isganaitis et al. [103] found in the cord blood of children who later developed obesity low levels of some tryptophan metabolites, including serotonin, betaine, and tryptophyl-leucine, and of the two methyl donors, dimethylglycine and N-acetylmethionine. However, despite nominally significant levels, these changes did not pass the false discovery rate (FDR) correction. Even the metabolites highlighted in cord blood by Hellmuth et al. [104], in 2017, did not show any significant association with postnatal measures after correction of multiple tests, contrary to what was observed for many metabolites and birth weight. Instead, Sorrow et al. [105] showed high levels of some lipid species in the cord blood of cases, including metabolites of acetaminophen and acylcarnitines, although no correction was applied for multiple tests. Regarding the metabolomic profile of plasma during early childhood, Rzehak et al. [106] analyzed plasma samples collected at the age of 6 months from 726 infants participating in a European multicenter study (Childhood Obesity Programme, CHOP), randomized to a high- or low-protein formula and breastfed. LPC at C14:0, associated with rapid growth in the first 6 months of life, after adjusting for feeding group as metabolic signals were significantly different between groups, was found to also be predictive of subsequent overweight at the age of 6. This result supports the existence of a metabolically programmed effect of childhood weight gain on subsequent obesity risk [86]. Similar work was conducted by Fleddermann et al. [107], who studied the plasma metabolomic profile of 4-month-old infants enrolled in a randomized control study to analyze the impact of low-protein infant formula on growth trajectories. Additionally, in this case, it was necessary to adjust according to the power group; however, no metabolites were predictive of BMI at 4 years, although six metabolites (asparagine, lysine, methionine, phenylalanine, tryptophan, and tyrosine) were positively associated with change in weight z-score for age and a metabolite (tyrosine) positively associated with change in BMI z-score for age between 1 and 4 months.

In the very recent systematic review of the literature [86], seven studies examined the effect on the metabolome of an intervention aimed at weight loss. Two studies were conducted by Leal-Witt et al. [108,109] on a Spanish cohort of 35 prepubertal obese children (7–10 years) before and after a lifestyle intervention that resulted in the reduction of BMI standard deviation score (BMI-SDS) from 3.56 to 3.11 and which has favored the adoption of a healthier food routine. The principal component analysis (PCA) conducted on the untargeted plasma profile (LC-MS) [108] highlighted a component (PC1) significantly altered by the intervention, closely related to sphingolipid metabolism. In fact, this intervention led to a decrease in numerous sphingolipid metabolites, including MS, ceramide, glycosyl-sphingosine, and sulfatide; results, however, correlated with the improvement in the levels of glycated hemoglobin (HbA1c) recorded after the intervention and not with the BMI values. The change in metabolites detected by urine NMR analysis (decrease in trimethylamine N-oxide (TMAO), 3-hydroxyisovalerate, and dimethylglycine and increase in xanthosine) was also not related to BMI value [109]. Pathmasiri et al. [110] conducted an exploratory investigation to determine the relevance of using metabolomics in providing noninvasive markers to predict an individual’s responsiveness to a 3-week intervention program. They also integrated psychosocial and metabolomic profiles to predict an adolescent’s positive response to weight loss. This study was conducted on obese adolescents aged 12–18 with a BMI < 95th percentile. Subjects were classified as responders (≥0.5 unit BMI decrease) or nonresponders (≤0.5 unit BMI decrease) at intervention based on weight loss. They also analyzed psychosocial variables, such as self-esteem and depression, which were shown to be independent of weight loss. Multivariate analysis of urine NMR spectrum highlighted a specific pattern that discriminates responders from nonresponders; specifically, lower levels of 2-oxoisocaproate, resulting from incomplete breakdown of BCAAs, were predictive of weight loss, while lower concentrations of tyrosine, taurine, and glycine characterized the decrease in BMI. Subsets of metabolites were also identified that were best associated with impaired self-esteem and depression, demonstrating that integrating metabolomic and psychosocial data can provide a profile of biochemical (metabolites) and psychosocial (self-esteem and depression) markers relevant to determine the total response to the treatment of an individual. Short et al. [111] enrolled American Indian boys and girls, aged between 11 and 17 years, with obesity (Ob, *n* = 58) or normal weight (NW, *n* = 36), and evaluated the impact of a 48-week training intervention (3 consecutive 16-week phases). Preliminary analysis showed lower aerobic fitness and insulin sensitivity along with 17 higher AA and 7 lower AA in the Ob group. Among the increased AAs found were BCAAs (+10%–16%), ArAAs (+15%–32%), and glutamate, all positively correlated with body fat and negatively with insulin sensitivity. Specifically, a metabolite of lysine, 2-aminoadipic acid (2-AAA), and a metabolite of valine, β-aminoisobutyric acid (BAIBA), were found to be 47% higher and lower, respectively, in the Ob group, showing a positive correlation for 2-AAA and negative for BAIBA with insulin sensitivity. The exercise intervention program increased aerobic fitness by 10%, but body composition, insulin sensitivity, and AAs showed no significant changes. In a 1-year lifestyle intervention study of 80 obese children aged 6–15 years, 40 children achieved a significant reduction in BMI-SDS (≥0.5), while 40 did not improve their condition (<0.1). From targeted metabolomics mass spectrometry analysis, lower serum long-chain unsaturated phosphatidylcholine concentrations and lower waist circumference emerged as the most significant predictors of BMI-SDS reduction during intervention, both through univariate regression models compared with the multivariate approach with a selection operator and minimum absolute contraction (LASSO) [112]. The analysis of 14 metabolites, which emerged to be related to obesity in previous studies, was conducted by Reinehr et al. [113] on the serum of 80 obese children with a reduction in BMI-SDS ≥ 0.5 compared with 80 obese children, which reported no weight changes consistent with a 1-year lifestyle intervention. In this case, in children who did not show weight changes, no significant changes in the concentrations of metabolites were found, while in children with a substantial weight loss, glutamine; methionine; lysophosphatidylcholines LPCaC18:1, LPCaC18:2, and LPCa20:4; and acyl-alkyl-phosphatidylcholine PCaeC36:2 increased significantly. In contrast, C12:1 and C16:1 acylcarnitines; proline; and phosphatidylcholines PCaeC34:1, PCaeC34:2, PCaeC34:3, PCaeC36:3, and PCaeC38:2 did not undergo significant changes. The same group [114] conducted a longitudinal study on the changes of steroid hormones in 40 obese girls aged between 13 and 16 years, of which 50% had polycystic ovary syndrome (PCOS) adhering to a lifestyle intervention lasting 1 year. Preoperatively, obese girls with PCOS showed significantly higher androstenedione and testosterone concentrations than obese girls without PCOS, while other steroid hormones, including glucocorticoids, mineralocorticoids, estrogens, and androgen precursors, showed no significant changes. Weight loss following the intervention program resulted in a significant decrease in the concentrations of testosterone, androstenedione, DHEA-S, cortisol, and corticosterone in obese girls with PCOS. Hormonal changes in obese non-PCOS girls were slightly different, with only significant decreases in DHEA-S, cortisol, and corticosterone concentrations being observed. Steroid hormones did not change in the absence of weight changes, except for an increase in estradiol in obese PCOS girls.

## 6. Compared Metabolomics and Microbiomics Analysis of Childhood Obesity

The studies concerning the application of metabolomics and microbiomics in the investigation of childhood obesity are summarized in Table 1.

Mastrangelo et al. [17] performed the first study, where a comprehensive untargeted metabolic fingerprinting protocol was applied to analyze metabolic pathways potentially associated with IR in prepubertal obese children. From the analysis of 60 prepubertal obese children (30 girls/30 boys, 50% IR and 50% non-IR in each group, but with similar BMIs), the involvement of three main factors was highlighted: the previously discussed CCM, inflammatory homeostasis, and the microbiota, supporting the multifactorial nature of the interaction between obesity and IR. The alteration of the CCM can also have important repercussions at the brain level. In fact, the increase in ArAAs, precursors of serotonin (from tryptophan) and catecholamines (from phenylalanine and tyrosine), competes with the BCAAs for transport into the cells. Consequently, their increase in serum may be caused by an alteration of their transport in the brain determined by the excess of BCAAs. The alteration of the inflammatory homeostasis was highlighted by the exacerbation, in the presence of hyperinsulinemia, of the imbalance of the proportions between proinflammatory effectors, determined by the elevated levels of lysophospholipids (LPs), in particular the lysophosphocholine subgroup, and anti-inflammatory, with decreases in bilirubin, nitro-octadecenoate, docosahexaenoate, and docosapentanoate. As far as the microbiota is concerned, the most marked changes of the analyzed metabolites concern secondary bile acids (BA), glycodeoxycholate, and taurodeoxycholate, significantly increased in obese subjects with IR compared with their noninsulin resistant counterparts. The secondary BA values at the plasma level support the important contribution of the microbiota at the metabolic level. In fact, their presence is determined by the action of the microbiota, responsible for the transformation of the cholate, conjugated with glycine or taurine, into secondary BAs, reabsorbed by the distal ileum via the enterohepatic circulation. These molecules behave as signal molecules, able to regulate the homeostasis of lipids and glucose. Further confirmation of the role of the microbiota in metabolic pathologies is the increase in the levels of piperidine, a by-product of the degradation of amino acids mediated by the microflora.

Troisi et al. [115] focused on the exploration of the interaction between the urinary metabolomic signature studied with GC–MS and gut–liver axis (GLA) abnormalities in pediatric obesity and nonalcoholic fatty liver disease (NAFLD). They analyzed both intestinal permeability (PI) and small intestinal bacterial overgrowth (SIBO) together with insulin resistance in 36 children aged 5 to 16 years, including 22 who were obese (10 without NAFLD and 12 with NAFLD). SIBO was increased in all obese children, while PI was increased preferentially in those with NAFLD. Analysis of the urinary metabolome highlighted the presence of alterations affecting numerous metabolites that contribute to delineating a metabolomic footprint of numerous obesity-related metabolic alterations. Furthermore, the metabolic pathways involved were closely related to the observed alterations in GLA, such as PH and SIBO. Low levels of xylitol and phenylacetic acid (PAA) together with high glucose levels discriminated against obese children. Both the low levels of xylitol and PAA are indicators of incorrect eating habits, while the high levels of glucose, in agreement with the data present in the literature, from the pathological point of view represent the confirmation of the higher incidence of insulin resistance in these subjects. In obese subjects, in particular those with NAFLD, a higher concentration of 1-methylhistidine was also detected, probably associated with an incorrect diet, typically Western diet, or as underlined by the authors, it can also derive from an increased oxidation in the skeletal muscle, a commonly reported condition in obesity-related pediatric NAFLD due to deficiencies in antioxidant stores. Regarding the impact of the microbiota, an increase in urinary p-cresylsulfate (PCS), an intestinal microbial metabolite deriving from the secondary metabolism of p-cresol, was observed in obese children without NAFLD. Data present in the literature [116] support that the decrease in its levels is associated with a higher intake of fibers and a low consumption of meat, confirming the inverse correlation observed between PCS and the 1-MHis metabolite. A correlation between the metabolic pathways of BCAAs and/or their metabolites has also been observed not only with excess visceral fat (leucine/oxovalerate) but also with a greater alteration of IP and SIBO (valine metabolites).

**Table 1:** Metabolomics and microbiomics studies.

Authors/Year	Patients	Samples	Technique	Main Metabolomics Findings	Microbiota Influence	Clinical Significance
Mastrangelo et al. [17] 2016	60 prepubertal OB children (30 girls/30 boys, 50% IR and 50% non-IR in each group, with similar BMIs)	Serum	LC–MS, GC–MS, CE–MS	IR group: ↑ LPs (), BCAAs, ArAAs, alanine, proline, pyruvate, ketoisocaproic acid, C3 and C4 acylcarnitines, ↓ free carnitine, bilirubin, nitro-octadecenoate, docosahexaenoate, docosapentanoate, and 3-hydroxybutyrate	IR group: ↑ glycodeoxycholate, Taurodeoxycholate, and piperidine	Metabolic pathways inherent to inflammation, central carbon metabolism along with some metabolites from the gut microbiota were more altered in obese children with IR, with alterations more pronounced for the female sex
Troisi et al. [115] 2017	36 children/adolescents (aged 5–16 years), 22 OB (including 10 without NAFLD and 12 with NAFLD)	Urine	GC–MS	In obese group: ↑ levels of glucose/1-methylhistidine ↓ levels of xylitol, phenyl acetic acid, and hydroquinone leucine/oxovalerate correlated with excess of visceral fat centimeters valine metabolites correlated with more deranged IP and SIBO	↑ urinary PCS (an intestinal microbial metabolite) in obese children without NAFLD urinary PCS correlated negatively with the presence of SIBO	A complex network of urinary molecules appears to be correlated with clinical phenotype and distinguishes obese children between those with and without NAFLD. Individual or grouped metabolites interact with anthropometrics and variously aggregated GLA parameters
López-Contreras et al. [68] 2018	138 unrelated children, 67 HWC and 71 OB (80 boys and 58 girls, aged 6–12 years)	Serum + Stool	FIA–MS + 16sRNA	↑ serum levels of BCAA (valine and leucine/isoleucine) and ArAAs (phenylalanine and tyrosine) in obese phenylalanine serum levels show a negative and significant correlation with both B. plebeius and unclassified Christensenellaceae abundance	No significant differences in phyla abundances or Firmicutes/Bacteroidetes ratios ↑ Bacteroides eggerthii abundance in obese that correlated positively with body fat percentage and negatively with insoluble fiber intake ↑ Bacteroides plebeius and unclassified Christensenellaceae abundances in normal weight	Identification of bacterial species associated with obesity and related metabolic alterations in order to design dietary intervention studies, which could eventually lead to translational dietary recommendations
Quiroga et al. [117] 2020	43 children (aged between 7 and 12 years), 29 OB and 14 HWC. OB group was randomly split into two categories (20 training participants followed a 12-week combined strength and endurance training program; the control obese group, 9, maintained their normal daily routines)	Stool	H^1^ NMR + BaseSpace Application 16 S Metagenomics v1.0 (Illumina Inc.)	exercise intervention modified the metabolic profile in obese patients, representing a dispersing factor: ↓ BCAAs (isoleucine and leucine) and xylose, glucose, and galactose moderate ↓ formate and alanine	In obese: no significant differences in phyla abundances or Firmicutes/ Bacteroidetes ratios ↑ phylum Proteobacteria ↓ genera Clostridium, Bifidobacterium, Coprococcus, Akkermansia, and Streptococcus ↑ Bacteroides, Prevotella, Phascolarctobacterium, and Paraprevotella exercise intervention: ↓ Proteobacteria phylum and Gammaproteobacteria class ↑ genera Blautia, Dialister, and Roseburia	Identification of an obesity-related deleterious microbiota profile that is positively modified by physical activity intervention
Jaimes et al. [91] 2021	52 children (aged 7 to 16 years), 16 HWC, 17 HW, 19 OB	Stool	H^1^ NMR + 16S rRNA	↑fecal butyrate in the OB compared with the N group ↑ arabinose and galactose in OW and OB (strong positive correlation with each other, and both showed a significant positive correlation with the BMI z-score) ↑ TMA in the OW and OB	↓*Escherichia* in relative abundance from the N to the OB group (genus includes both commensal and pathogenic species) ↑ *Tyzzerella* subgroup 3 in a relative abundance from the N to the OB group	Increased energy harvest in OB by the human gut microbiota
McCann et al. [118] 2021	54 adolescents (aged 10–18 years), 27 with BMI ≥ 95th percentile and 27 HWC. OB group are patients in the Healthy Lifestyles program, which includes visits to a multidisciplinary clinic and membership in a community-based fitness program (6 months of intervention)	Serum + stool	*ISQ single quadruple GC*–*MS* + UPLC/MS–MS + 16S rRNA	after FDR adjustment for multiple comparisons, no metabolites were significantly different between the OB and HWC groups nominally significantly different in OB: ↑ BCAA valine ↓ KIC and KMV	Significant differences in measurements of alpha and beta diversity between OB and HWC group 2 Lachnospiraceae families and a Lachnospira species characterized OB samples while members of the Christensenellaceae, Ruminococcae UCG_14 families and Alistipes species defined HWC	Suggestion of a metabolic signature of obesity unique to adolescents and confirmation of a metabolic and microbiome markers of obesity

Abbreviations: BCAAs, branched-chain amino acids; ArAAs, aromatic amino acids; PCS, p-cresylsulfate; HWC, healthy weight control; OB, obese; HW, overweight; KIK, α-ketoisocaproate; KMV, α-keto-β-methyl valerate; NAFLD, nonalcoholic fatty liver disease; SIBO, small intestinal bacterial overgrowth; GLA, gut–liver axis; TMA, trimethylamine; GC, gas chromatography; MS, mass spectrometry; UPLC, ultra performance liquid chromatography; CE, capillary electrophoresis; LC, liquid chromatography; FIA, flow injection analysis; H^1^ NMR, proton nuclear magnetic resonance.

López-Contreras et al. [68] performed a comparative analysis between the composition of the intestinal microbiota in obese children and serum amino acid levels together with obesity-related metabolic parameters, with a view to identifying bacterial species associated with obesity and its correlation with metabolic alterations to design personalized dietary intervention studies. The abundance of intestinal microbial taxa was investigated by 16S rRNA sequencing, and serum amino acid levels were measured by mass spectrometry in 67 normal weight and 71 obese children. As previously discussed, the analyses did not reveal significant differences in the abundances of the phyla or in the Firmicutes/Bacteroidetes ratios between the two groups; nevertheless, the obese children were characterized by a greater abundance of Bacteroides eggerthii. This abundance was positively correlated with body fat percentage and negatively with insoluble fiber intake. Conversely, children of normal weight were distinguished by a greater abundance of Bacteroides plebeius and unclassified Christensenellaceae, which correlated negatively with serum phenylalanine levels.

Quiroga et al. [117] analyzed 43 children (7–12 years), of whom 29 were obese and 14 were healthy controls without signs of pubertal development. The obese pediatric group was randomly divided into two categories, depending on whether or not the participants submitted to the training protocol: 20 participated in the 12-week training program which combined both strength and endurance, while the 9 subjects in the obese control group maintained normal daily routines. Gene sequencing analyses revealed the presence of an obesity-associated bacterial profile characterized by the prevalence of the phylum Proteobacteria. Additionally, in this case, no significant differences were found in the distribution of Firmicutes and Bacteroidetes between the two groups. However, analyses at the genus level showed significant differences: a decrease in Clostridium, Bifidobacterium, Coprococcus, Akkermansia, and Streptococcus genera characterized obese children. Adherence to the training program substantially changed the relative abundance of some species, such as Clostridia, Flavobacteriia (Bacteroidetes phylum), Actinobacteria, and Gammaproteobacteria, among obese children. In fact, the microbiota profile at the class level tended to be similar to the healthy control group in the children who underwent the program. The application of a PLS-DA method to the H^1^-NMR analysis at the start of the study showed a clear cluster formed by the metabolites of all obese patients compared with healthy children who were missing. Instead, adherence to the training program represented a dispersion factor. In fact, the metabolic profile of the obese children adhering to the intervention underwent significant changes, with a decrease in branched-chain amino acids, such as isoleucine and leucine, xylose, glucose, and galactose and a modest decrease in other metabolites, such as formate and the alanine. Additionally, in this study, a comparative analysis was performed between the different microbial compositions of the intestine and the related fecal metabolites, which highlighted statistically significant interactions between 11 metabolites and 8 bacterial genera. A negative correlation was observed between Alkaliphilus and Clostridium, both reduced in obese patients after exercise, with fecal glutamate. A negative correlation also emerged between the genera Lachnospira, Veillonella, Roseburia, and Blautia, reduced in obese patients and increased by training, with the fecal metabolites p-cresol, caprate, isovalerate. The same three metabolites were instead positively correlated with the genera Oscillospira and Flavobacterium. Instead, these two genera showed a negative association with glucose. Furthermore, Roseburia also showed positive correlation with acetate and nicotinate, while Oscillospira showed positive correlation with propionate and negative with nicotinate, succinate, and lysine.

Jaimes et al. [91] sought to identify potential metabolic and fecal bacterial signatures that allow identification of overweight/obesity status in children/adolescents. Through H1 NMR analysis and 16S rRNA sequencing, the fecal metabolic profile and bacterial composition of 52 children (7–16 years), including 16 with normal weight, 17 overweight, and 19 with obesity, were studied. Metabolomics analysis identified four metabolites that were significantly different between study groups: arabinose, butyrate, galactose, and trimethylamine. Arabinose and galactose increased in both the obese and overweight groups. They also showed a strong positive correlation with each other, and both showed a significant positive correlation with BMI z-score. Trimethylamine (TMA) was also increased in obese and overweight patients, while butyrate was increased only in obese subjects. At the level of the microbiota, however, statistically significant differences emerged for two genera, Escherichia (both commensal and pathogenic species) and Tyzzerella subgroup 3. In fact, a decrease in the relative abundance of Escherichia and an increase in the relative abundance of the genus Tyzzerella subgroup were recorded from children of normal weight compared with those who were obese. However, there was no significant difference in alpha-diversity between the three study groups and no significant correlations were found between significant taxa and metabolites. The authors conclude that the observation of increased fecal butyrate in overweight/obese children and some monosaccharides in feces support the hypothesis of increased energy production in obesity by human gut bacteria.

McCann et al. [118] performed a metabolomics analysis in conjunction with 16S rRNA sequencing to create a biorepository of clinical, metabolomics, and microbiome samples from adolescents with obesity as they underwent lifestyle modifications lasting 6 months. Fifty-four adolescents (10–18 years of age) were analyzed, of which 27 had BMI ≥ 95th percentile and 27 had normal weight. The obese group consisted of patients participating in the Healthy Lifestyles program, which included visits to a multidisciplinary outpatient clinic and membership in a community-based fitness program. In the metabolomics analysis after FDR adjustment for multiple comparisons, no metabolites were found to be significantly different between the two groups. Nonetheless, some metabolites showed a nominal significant difference: increase in valine (BCAAs) and decrease in glycine in the obese group while the ketoacidic products of BCAA catabolism, such as α-ketoxocaproate (KIC) and α-keto -β-methylvalerate (KMV), showed an opposite trend. Higher values of serum glycerol and insulin also characterized the obese group. Regarding the microbiome analysis, significant differences emerged in the measurements of alpha- and beta-diversity between the two groups, two families of Lachnospiraceae and one species of Lachnospira characterized the samples of the obese, while members of the families Christensenellaceae, Ruminococcae UCG_14, and species of Alistipes defined controls. Further analyzes were also conducted to identify microbiome community configurations that may be associated with obesity. The application of the Phylogenetic Isometric Log-Ratio (PhILR) transformation aimed at selecting the most effective taxonomic equilibria in predicting the patient’s belonging to the obesity or control group has shown that in normal weight subjects, the abundance of Eubacterium brachy genera compared with the abundance of the AD3011 Family XIII group was higher than in the obese. Furthermore, a lower abundance ratio of members of the Lachnospiraceae family compared with Ruminococcus gnavus was found in obese subjects. A further phylogenetic analysis was conducted this time without taking weight status into account. Five phylogenetic clusters emerged, which were then subsequently associated with BMI, highlighting how some clusters were more likely to appear in the obese cohort than in the normal weight and vice versa. In fact, a decrease in the abundance of members of the Bacteroides family and an increase in the abundance of several Ruminococcae and specific members of Prevotellaceae were most likely found in the obese group.

## 7. Conclusions

As a complex medical problem resulting from the interaction of physical and environmental factors, childhood obesity has become a global pandemic in developed countries. Prevention together with early intervention in the pediatric age are also important strategies to counteract the persistence of obesity in adulthood, with a consequent increase in morbidity and mortality. Furthermore, it can be seen that obesity is such a complex disease that it affects the body as a whole, with consequent numerous metabolic and psychological alterations, sometimes interrelated.

The evidence supporting the alteration of the microbiota as a critical factor in the etiology of obesity is abundant, although still not completely defined and understood. Indeed, microbiomics, through careful analysis of gut microbiota composition, could become one of the potential treatments for obesity, although further research is required in order to validate not only the effectiveness but also the efficiency of this strategy (e.g., dietary or lifestyle intervention programs, probiotic supplement).

The findings in the literature regarding the alterations of the metabolome in obese children appear clear, amply highlighted by the metabolomics studies conducted up to now. In fact, the existence of differentiated metabolic profiles in obese children has emerged, characterized by the presence of characteristic metabolites, indicators of the dysregulation of particular metabolic pathways almost totally attributable to a central disorder, i.e., the IR. Therefore, the identification of specific pathology-related biomarkers and the elucidation of altered metabolic pathways through metabolomics could allow us to better characterize the biological behavior of a system in response to external stimuli. This enables us to clarify the complex network of interactions between nutrients and molecules highlighting the individual metabolic response to dietary treatments, with the aim of devising a type of diet tailored to the genes of each individual.

In any case, although some limitations can be deduced from the published systematic reviews, such as a reduced number of samples and the scarcity of studies on the matter, some metabolites, in particular those belonging to the metabolism of lipids and amino acids, could be attributed a role in the future risk of developing metabolic diseases. In fact, the nutritional status in early childhood affects metabolic processes and health throughout the life span.

However, not only the current absence of internationally agreed reference measures for normal insulin sensitivity in childhood but also the lack of specific metabolomic databases to classify predictive biomarkers in early risk stratification does not yet allow the timely detection of high-risk phenotypes. This is combined with the necessity of further research due to the lack both of longitudinal studies, for the evaluation of the predictive potential of the metabolites, and of intervention, functional to the evaluation of the reversibility of the pathology and the effectiveness of personalized intervention measures.
